# Validity and reproducibility of a web-based dietary assessment tool: a cross-sectional study in an adult Danish population

**DOI:** 10.1017/jns.2025.10010

**Published:** 2025-06-04

**Authors:** Sadime Basak Kisi, Caroline Filskov Petersen, Rikke Sand Andersen, Sidse Ida Ingemann Rasmussen, Alexandr Parlesak, Sine Højlund Christensen, Hanne Lysdal Petersen, Nina Rica Wium Geiker, Mette Friberg Hitz, Inge Tetens

**Affiliations:** 1 Department of Nutrition, Exercise and Sports, University of Copenhagen, Copenhagen, Denmark; 2 Medical Department, Zealand University Hospital, Nykøbing F, Denmark

**Keywords:** Biomarker, Folate: myfood24^®^, Protein, 7-day weighed food record

## Abstract

This repeated cross-sectional study assessed the validity and reproducibility of the myfood24^®^ dietary assessment tool against dietary intake biomarkers in healthy Danish adults. The study included 71 healthy adults (14/57 m/f), aged 53.2 ± 9.1 years with an average BMI of 26.1 ± 0.3 kg/m^2^. Participants were instructed to complete seven-day weighed food records using myfood24^®^ at baseline and 4 ± 1 weeks thereafter. Estimated mean dietary intake was compared with objective measures of energy metabolism and selected dietary intake biomarkers in fasting blood (folate) and in 24-hour urine (urea, potassium). Resting energy expenditure was measured by indirect calorimetry. Application of the Goldberg cut-off classified 87% (*n* = 62) of participants as acceptable reporters. A strong Spearman’s rank correlation was observed between total folate intake and serum folate (*ρ* = 0.62). Acceptable correlations were noted for serum folate (*ρ* = 0.49) and urinary potassium excretion (*ρ* = 0.44) with estimated and measured protein intake (*ρ* = 0.45); energy intake and total energy expenditure (*ρ* = 0.38); potassium intake and potassium excretion (*ρ* = 0.42); and estimated fruit and vegetable intake. Reproducibility analysis revealed strong correlations (*ρ* ≥ 0.50) across most nutrients and food groups, except for fish and vitamin D (ρ = 0.30 and *ρ* = 0.26, respectively). Notably, reproducibility for folate and total vegetable intake exhibited the highest correlations (*ρ* = 0.84 and *ρ* = 0.78, respectively). In conclusion, while some limitations exist, myfood24^®^ remains a useful tool for ranking individuals by intake, particularly in studies focusing on relative comparisons.

## Introduction

Dietary assessment tools are used to estimate habitual diet, relate dietary data to health/disease outcomes, and to assess compliance with dietary guidelines and associations with nutritional status. In the context of dietary assessment, validity is the extent to which the tool documents the intake of all food items precisely and accurately during the registration period and produces reliable values for the associated nutrient intake.^(1–4)^ However, self-reported dietary assessments may have several limitations, including recall bias,^(2,3)^ misreporting of energy intake (EI), and social desirability bias.^(3)^ Additionally, errors independent of self-reported methods may arise from food composition databases (FCDB), which cannot fully account for the natural variation in nutrient content. Further inaccuracies may be introduced during database generation due to factors such as sampling design, analytical methods, and nutrient labelling.^(5)^ These errors, combined with those of dietary assessment methods, can cumulatively impact the estimation of energy, food, and nutrients. Additionally, when relative validity is assessed by comparing one dietary assessment method to another, good agreement between the test and reference methods does not necessarily indicate that the method provides objectively valid results as each method may have shortcomings.^(5)^ Seven-day weighed food records (7-day WFR) are generally regarded as the best method for obtaining accurate and reliable estimates of dietary intake.^(2,4)^

Validation studies of dietary assessment methods have used dietary intake biomarkers as reference instruments,^(5–7)^ assuming they provide objective measures of true intake.^(8)^ However, most biomarkers are relative measures influenced by factors such as metabolism, inter-individual variability,^(2,4,6,9)^ and food matrix effects.^(9)^ To avoid bias, errors in test and reference methods should ideally be independent of each other.^(5,8)^ This is particularly important when combining dietary recall methods with biomarkers, as correlated errors can distort dietary intake estimates.^(1,3,7)^

Reproducibility reflects the consistency of a method in producing similar results when applied repeatedly to the same individuals over time. Reproducibility indicates reliability and precision, which are key in limiting random errors and supporting the validity of the data collected.^(10,11)^ Previous reproducibility studies have repeated dietary assessments 1 week to 2 years apart.^(10)^ One week is the minimum recommended interval between repeated measurements as respondents may recollect and repeat their answers if the interval is shorter, artificially enhancing repeatability,^(10)^ whilst dietary registrations are less reproducible over longer periods due to genuine changes in habitual dietary intakes and random fluctuations.

Technology-based dietary assessment tools, such as web-based self-administered 24-hour dietary recall (24HDR), offer promising solutions to the challenges of traditional dietary assessment methods.^(12,13)^ These tools can reduce the burden in effort, time, and cost, enhance data quality by standardising processes, and be adapted for use in various populations and countries.^(14,15)^ However, technological tools do not eliminate all limitations. Challenges such as misreporting and recall bias may persist and require further validation to ensure accuracy and reliability in diverse settings.^(12)^ Myfood24^®^ is a fully automated online dietary assessment tool initially developed for the UK population. It supports both self- and interviewer-administered 24HDR or food records (FR) and includes user-friendly features such as support on determining portion size, helpful food and meal images, pop-up windows for commonly forgotten foods, a recipe builder, and a help function with detailed texts and videos. Its web interface enables researchers to customise the tool for use in diverse project designs.^(13,14,16,17)^

Myfood24^®^ has been shown to be practicable in various groups, including women with gestational diabetes, adolescents, adults, and the elderly, and for repeated short-term applications.^(13,14,16)^ It has been adapted for use in countries other than the UK. However, the adaptation process involves changes, particularly in the underlying food composition databases, which may affect functionality, usability, and accuracy.^(14,15)^ Myfood24^®^ has been validated for British adults,^(18)^ British adolescents,^(17)^ and German adults.^(15)^ However, the validity of each version of myfood24 modified for use in other populations must be assessed.^(15)^

In this study, we selected biomarkers based on their established use in dietary validation research and relevance to health. Commonly used biomarkers include indirect calorimetry, doubly labelled water, urinary nitrogen and potassium, and plasma folate.^(19–24)^ Energy and protein intake were assessed due to their metabolic significance and folate was included as a key indicator of fruit and vegetable intake, particularly leafy greens, which are central to dietary guidelines for health and disease prevention.

By considering these factors, we aim to contribute to the ongoing efforts to refine dietary assessment tools and improve their applicability in research. The objective of the present study was to assess the validity and reproducibility of myfood24^®^ using dietary intake biomarkers in a group of healthy Danish adults.

## Methods

### Subjects and study design

**Fig. 1.**
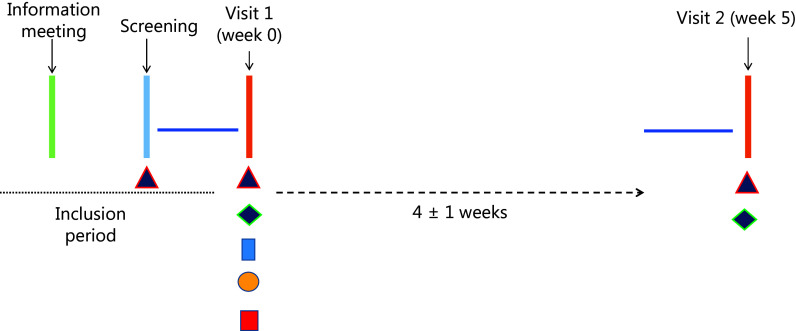
Study design overview. The figure outlines the study timeline. During the inclusion period, participants attended an information meeting, followed by screening for eligibility. At Visit 1 (week 0), measurements included height and weight (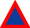), fasting blood samples (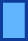), 24-hour urine sample hand-in (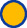), and indirect calorimetry (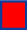). Participants completed a 7-day WFR 1 week prior to both visits (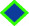), and handed these in (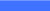) on both visits. Anthropometric measurements were repeated at Visit 2 (week 5) after a 4 ± 1 week interval.

This is a repeated cross-sectional validation study of a self-administered web-based dietary assessment tool, myfood24^®^, with a 7-day WFR repeated after 4 weeks. The study included an information meeting, a screening visit and two visiting days: Visit #1 (V1) at baseline and Visit #2 (V2) in week 5 (Fig. 1). The study was conducted between October 2022 and March 2023. Sample analyzes, data processing, and reporting were completed by January 2024.

The study protocol was approved by The Danish Scientific Ethics Committee for the Capital Region (Journal- no.: H-22030420), and the study was registered in ClinicalTrials.gov with the number NCT05600530.

Participants were eligible if they were aged between 35 and 70 years, self-reportedly healthy, had a BMI between 22 and 32 kg/m^2^, were fluent in Danish, had regular access to high-speed internet, and had a valid email address. Participants also had to be weight stable over the previous three months (defined as no weight gain or loss above 2.5% of their body weight) and had to be willing to continue their current dietary habits and physical activity levels throughout the study period. Exclusion criteria were: history or diagnosis of any chronic disease (e.g. diseases of the heart, liver, or kidney), current use of medication affecting body weight, lactating, pregnant or planning to become pregnant within the study period and being engaged in elite sports or similar strenuous exercise ≥5 h/week. Dietary patterns interfering with the study protocol, and/or the use of dietary supplements or medication that may affect the exposure biomarkers, as judged by the investigator, were also exclusion criteria.

### Recruitment and visits

Our subjects were recruited at the University of Copenhagen, Frederiksberg campus, by advertisement through social media channels and flyers, and from an internal volunteer list at the Department of Nutrition, Exercise and Sports.

At an introductory information meeting, candidates were informed about the inclusion criteria, study design and objectives, how to complete a 7-day WFR using myfood24^®^, and how to collect a 24-hour urine sample. Subjects were given time to consider participation.

A screening visit was arranged for candidates who agreed to take part in the study and signed the consent form. During the screening, participants were provided with a kitchen scale for accurate weighing of food items and were given instructions, bottles, and cooling elements for the collection of 24-hour urine samples. They were evaluated in relation to the inclusion/exclusion criteria, and anthropometric measurements (height and weight) were taken. They were instructed on how to complete the dietary registration process in myfood24^®^.

At screening, participants were instructed to keep a 7-day WFR in myfood24^®^, collect a 24-hour urine sample on the final day of the WFR, and visit the study centre on the 8^th^ day (V1). At V1, they handed in their urine sample, fasting blood samples were taken, energy expenditure was measured using indirect calorimetry, they were weighed, and their height was measured. Participants completed another 7-day WFR using myfood24^®^ within 4 ± 1 weeks of V1 and returned to the study centre for a second visit (V2).

At V2, participants were offered a 1:1 consultation of a minimum of 20 minutes with a dietitian, where they were given feedback on their dietary habits compared to the official Danish dietary guidelines, focusing on selected food groups and on macronutrient contribution to total energy intake. All the feedback was based on results from the first 7-day WFR. The official Danish dietary guidelines were sent to all participants at the end of the study.

### Anthropometric measurements

Height was measured with a wall-mounted stadiometer (Hultafors) to the nearest 0.5 cm. Weight was measured twice on a TANITA MC 780 MA weighing scale^(25)^ to the nearest 0.1 kg and reported as the mean value of the two recordings. Body weight was measured at both V1 and V2 to control for maintenance of energy balance. It was assessed whether body weight at V2 deviated more than 2.5% from weight at V1.

### Dietary assessment and dietary intake biomarkers

#### Weighed food records

All candidates attending the introductory information meeting at the study centre (Department of Nutrition, Exercise and Sports, University of Copenhagen) were instructed both in writing and verbally on how to use myfood24^®^. They were introduced to and encouraged to use the pictures of portion sizes for estimations in myfood24^®^ when exact weighing was not possible. They were advised to contact project staff by email/message or call the project phone number with any further questions arising after the meeting. At the subsequent screening visit, all participants received digital kitchen scales (ProfiCook brand^(26)^) which measured in increments of 1 g up to a maximum weight of 5 kg. The participants then went on to complete a 7-day WFR on seven consecutive days, where they weighed and registered all food items consumed, including beverages and the intake of water. Participants who did not complete all seven days of the 7-day WFR were asked to register their intake on one additional day. If the missing day was the day immediately before V1, the participant was asked to register their intake on the day of V1. If the missing day was any other day in the course of the 7-day WFR week, they were asked to register their food intake on a day as soon as possible after V1.

The information obtained included the date of consumption, preparation method, and type and quantity of the food. Participants were also asked to register their intake of supplements by type, quantity, and brand name in myfood24^®^, and to mention them at study visits.

The myfood24^**®**^ Danish database was compiled by The Danish Cancer Society Research Center in 2017. It comprised 1668 adult and infant foods, including food items from the Danish Food Composition Table (FRIDA),^(27)^ the FCDB from Sweden (National Food Agency)^(28)^ and England FCDB (McCance and Widdowsons).^(29)^

If subjects could not find a specific food item in the myfood24^**®**^ food list, they took a photo of the item and sent it, together with a photo of the nutrition information label on the back of the packet, to the department. The information was then added manually by trained project staff to the subject’s 7-day WFR. Participants were also required to register recipes of homemade meals by weighing and recording all ingredients, as well as the amount consumed after preparation.

#### Collection and analysis of blood samples

Blood samples (20 ml) were collected at V1. After 30 min ± 5 min, the samples were centrifugated at 2754 × G for 10 min at 5°C. Serum was kept at –80°C until analysed. Folate was analysed in serum on an Immulite 2000 xpi competitive immunoassay system from Siemens Healthcare Diagnostics Ltd. with an internal serum pool with an average of 8.75 ng/ml and a CV of 6.05 %.

#### Collection and analysis of 24-hour urine

Participants were instructed to start 24-hour urine collection on the day before V1 — the last day of the first 7-day WFR — discarding the first morning urine and continuing to include the first urination the following morning. Participants received detailed written and oral information on the collection process. Participants were provided with appropriate collection devices to urinate into and were instructed to transfer all collected urine into the larger collection bottles provided. Participants recorded the time and date on the empty collection bottles before starting urine collection. The urine was kept cold in a cooling bag with frozen cooling bricks until delivery to the study site. The volume of the complete 24-hour urine samples was calculated based on weight and density. An aliquot of the urine was kept at –80°C until transportation to the Clinical Biochemistry Unit at the Zealand University Hospital for analysis.

Urinary potassium, creatinine and urea concentrations were measured using a Dimension Vista 1500 (Siemens AG, Munich, Germany) with the following coefficients of variation (CVs): serum concentrations of potassium (2.4% at low values, 1.2% at high values), and creatinine (5% at low values, 3% at high values).

Urea measured in the 24-hour urine sample was converted to total urinary nitrogen excretion (N) with the formula: total N per 24-hour, *g* = 0.028 (g/mol) × total urea per 24 hours, mmol. Urinary nitrogen values were then applied for the calculation of resting energy expenditure (REE).

Biomarker measurement of protein intake was calculated based on the excreted N, which was measured with a conversion factor of 6.25. We assumed that 81% of N and 80% of consumed potassium are excreted within 24 h.^(21,30)^

N values were calculated both with and without correction for creatinine excretion, the factor being based on an average daily excretion of creatinine of 21 mg/kg for males and 17.5 mg/kg for females, as given by Corder.^(31)^

#### Assessment of physical activity

Physical activity level was assessed using the short form of the International Physical Activity Questionnaire (IPAQ)^(32)^ at the screening visit. Metabolic Equivalents of Task units (MET), considered to be equivalents of calories burned during any physical activity, were used standardised for resting metabolic rate (RMR) as 1 MET = 3.5 ml/kg per min. They were obtained for three categories — strenuous, moderate and walking — from the IPAQ results.^(33)^ Hours, where no physical activity in these three categories or being seated were registered, were assumed to have been spent asleep or lying down and were registered with the corresponding MET values. Physical activity level (PAL) values were calculated as total MET value per 24 hrs. We removed participant data with a PAL above 2.5, as this or any higher value was considered unlikely to occur given our inclusion criteria.

#### Measurement of resting energy expenditure

Resting energy expenditure was measured by indirect calorimetry in a ventilated hood system (Oxycon Pro, Viasys Healthcare) for 2 × 25 min + calibration time on the day of V1. This method of estimating REE has previously been validated against estimates using DLW,^(6,34)^ the latter being the reference^(30)^ and gold standard^(35)^ for clinical settings. Participants reclined under a ventilated hood (canopy) in the supine position and rested for at least 20 minutes before the measurements began. Participants were told to lie still during the measurements and were not permitted to sleep, read or talk beyond brief communication with study staff. Two measurements were performed. Each measurement lasted for 25 minutes with 10 minutes of rest between measurements to allow stabilisation of oxygen consumption (VO2) and carbon dioxide production (VCO2). Resting energy expenditure was estimated from VO2 and VCO2 production in a given time using the equation by Weir^(36)^ and the constants of Elia and Livesey.^(37)^ Total energy expenditure results were obtained by multiplying the values from indirect calorimetry measurements with the PAL values obtained from the IPAQs. The assumption was that our participants were in energy balance and that the estimated TEE should therefore be similar to the calculated total energy intake.

#### Assessment of acceptable reporters

We used the Goldberg equation^(38)^ EI:BMR range of 1.05–2.28 to assess for acceptable reporters (ARs). Reporters with a ratio of less than 1.05 were characterised as underreporters (UR) while those with a ratio greater than 2.28 as overreporters (OR).

## Statistics

To consider the applicability of parametric tests, deviation from normal distribution was evaluated by the test of Shapiro Wilks. Most of the dietary variables were not normally distributed, even after log-transformation. Thus, all dietary variables are presented using median values and interquartile ranges, with non-parametric tests used for analysis. Spearman’s rank correlation test was applied to assess the correlation between estimated intakes and measured biomarkers. Additionally, simple and multiple linear regression analyses were conducted to assess the variance (R^2^) in biomarker data explained by dietary intake. Multiple models were adjusted for age and sex where relevant. Simple and multiple regression models were performed for both weekly average intake and intake on the day prior to Visit 1 (V1). Spearman’s ρ was calculated for the correlations between dietary intake biomarkers (serum folate, TEE, protein based on 24-hour urine N, potassium in 24-hour urine) and estimated intakes from myfood24^®^ for energy, protein, folate, and fruits and vegetables both for weekly average values and intake from the day before V1. Interpretation of correlation cut-offs were used with <0.20 being poor, 0.20–0.49 acceptable, and ≥0⋅50 being strong.^(39,40)^

The non-parametric Wilcoxon signed-rank test was applied to determine paired group differences between the first and second round of the averaged daily dietary registrations conducted over 7 d. The paired sample *t*-test was used to check for any differences in body weight between the two visits. The visual agreement between the two WFRs was displayed as proposed by Bland and Altman, plotting the difference between the two registrations versus the average of the measurements.^(41)^ Lower and upper limits, mean differences, and intra-class correlation coefficients were calculated using the statistical programme R, version R 4.3.2. Bland-Altman plots were generated in Microsoft Excel.

## Results

### Subjects

**Fig. 2.**
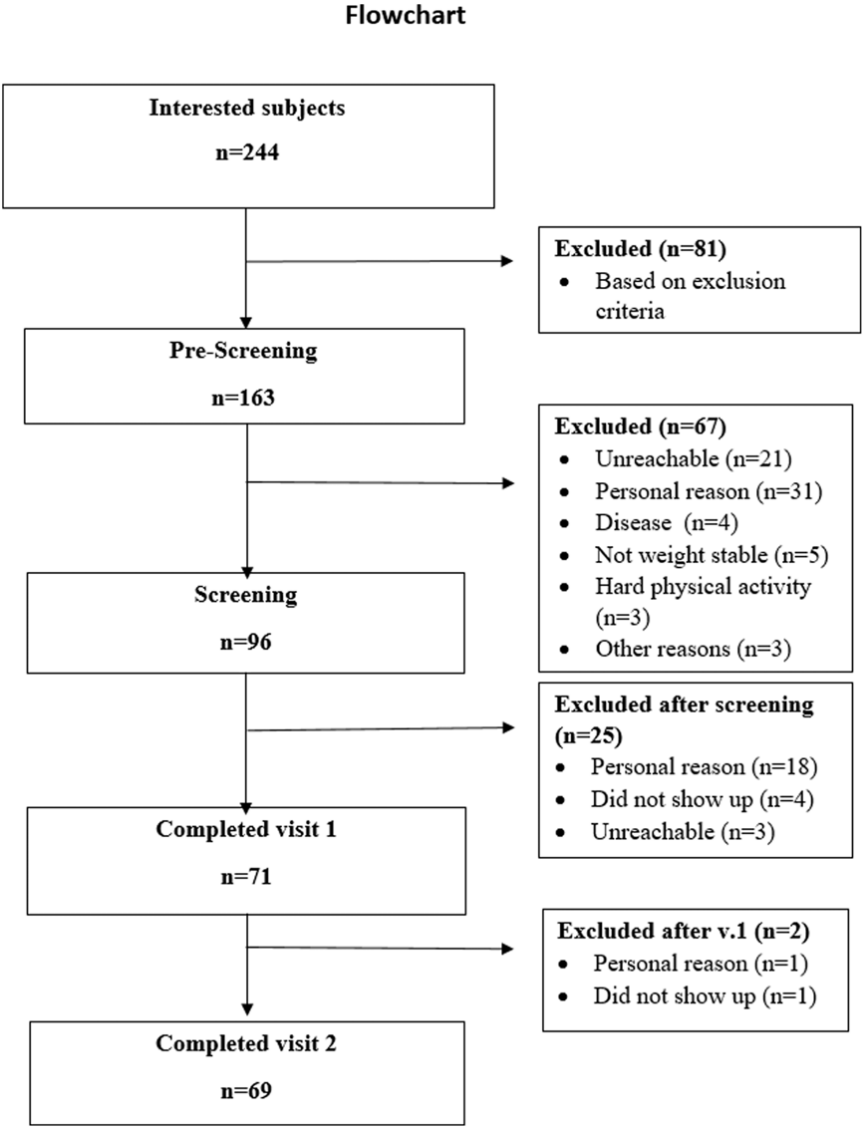
VALID flow chart. The flowchart illustrates the participant recruitment process. Of 244 subjects initially expressing interest, 163 were included in the pre-screening after 81 were excluded based on the exclusion criteria. Of the 163 individuals included in the pre-screening, 96 proceeded to the screening phase, and 67 were excluded for reasons such as being unreachable, personal reasons, or not meeting specific criteria. A total of 71 participants completed Visit 1. Two additional participants were excluded after Visit 1, leaving 69 participants who completed both visits.

Of the 244 subjects initially interested in participating in the study, 81 were excluded based on study criteria, resulting in 163 subjects proceeding to the pre-screening process (Fig. 2). Sixty-seven prospective participants were excluded at pre-screening, leaving 96 individuals eligible for screening. Subsequently, 71 participants (57 f/14 m) completed Visit 1. After Visit 1, two more participants were excluded, leaving a total of 69 participants (55 f/14 m) who completed both visits (Fig. 2). The baseline characteristics of the participants included in the analysis are presented in Table 1.

**Table 1.** Anthropometrics, smoking, alcohol consumption, and educational status of participants included in the VALID study mean + SD; *n* (%) (*n* = 71)

	All	Men	Women
Participants, *n* (%)	71	14 (20)	57 (80)
Age (yrs)	53 + 9.1	57 + 8.1	52 + 9.2
Mean weight (kg)	74.4 + 1.3	85.4 ± 2.7	71.7 ± 1.2
Mean height (m)	1.69 + 0.01	1.81 ± 0.01	1.66 ± 0.01
Mean BMI, kg/m^2^	26.1 + 0.3	25.9 ± 0.8	26.1 ± 0.3
Tobacco smoking, *n* (% by group)	3 (4.2)	0 (0)	3 (5.3)
Alcohol units per week, *n* (% by group)			
0–1	29 (41)	5 (36)	24 (42)
2–7	33 (46.4)	6 (43)	27 (47.3)
7–12	5 (7)	1 (7)	4 (7)
12–24	3 (4.2)	1 (7)	2 (3.5)
>24	1 (1.4)	1 (7)	0 (0)
Education level, *n* (% by group)			
Primary and lower secondary education	1 (1.4)	1 (7)	0 (0)
General upper secondary education	2 (2.8)	0 (0)	2 (3)
Vocational education	3 (4.2)	2 (14.2)	1 (2)
Short-cycle higher education	3 (4.2)	0 (0)	3 (5)
Medium-cycle higher education	33 (46.4)	3 (21.4)	30 (53)
Long-cycle higher education	25 (35.2)	5 (36)	20 (35)
PhD	4 (5.6)	3 (21.4)	1 (2)

There was no significant difference in body weight between the two visits (mean ± SD V1: 74.22 ± 10.89 and V2: 74.20 ± 10.90, *t* = 0.925, df = 70, *P* = 0.358). Applying the Goldberg cut-off method for assessing the number of subjects to be characterised as acceptable reporters (AR), UR, and OR, we found the proportion of AR to be 87% (*n* = 62), UR to be 13% (*n* = 9), and no OR. It was decided to keep the URs in the further analysis, as the aim of this study was to compare values extracted from myfood24^®^ with analysed biomarkers. One participant with a PAL above 2.5 was removed from the validity analysis.

### Dietary intake and validation

The average number of days completed in each WFR was 6.84, overall 6.91 for V1, and 6.76 for V2, with a mean time interval of 31.8 ± 6.8 days between the two registrations. Spearman’s rank correlation analysis revealed several significant correlations between dietary intake measures and the corresponding biomarkers (Table 2). Stronger correlations were found between dietary intake and urinary and blood biomarkers when expressed as the average weekly dietary values, compared to the dietary intake estimated from the day before biological sampling, as shown in Table S1.

**Table 2.** Spearman’s rank correlation coefficients with associated p values between measured biomarkers and estimated intakes from myfood24^®^ (daily average) among Danish adults participating in the VALID study

Estimated intake* from myfood24^®^	Dietary intake biomarkers	Spearman Rank Coefficient *ρ*	*P*-value
Energy (kcal/d)	Total energy expenditure (kcal/d)	0.27	0.02
Energy (kcal/d)	Resting energy expenditure (kcal/d)	0.44	<0.01
Total Protein (g/d)**	Protein assessed by 24-hour urine N (g/d)	0.46	<0.01
Total Folate (µg/d)**	Serum folate (µg /l)	0.62	<0.01
Dietary Fruit and Vegetable (g/d)	Serum folate (µg /l)	0.44	<0.01
Dietary Fruit and Vegetable (g/d)	24 hr urine K (mmol)	0.21	0.08
Dietary Potassium (mg/d)	24 hr urine K (mmol)	0.24	0.05

* Estimated daily intakes are calculated as the average of the 7-day WFR.

** Total intakes include both dietary intake and intake from supplements.

24-hr urine K, Potassium excreted in 24-hour urine.

There was an acceptable correlation between energy intake and both total energy expenditure (*ρ* = 0.27, *P* = 0.02) and resting energy expenditure (*ρ* = 0.44, *P* < 0.01). Regression models showed that energy intake explained 7% of the TEE variance using weekly averages and 9% using last-day intake (P = 0.027 and *P* = 0.008, respectively) (Supplementary Tables S5a and S5b). REE had stronger associations with energy intake, explaining 14% of the variance using weekly average and 11% using last-day intake (*P* = 0.004 and *P* = 0.008, respectively). After adjusting for age and sex in multiple regression models, REE explained 38% and 37% variance for weekly and last-day intake, respectively (both *P* < 0.05).

Dietary potassium intake accounted for 31.4% of the variation in fruit and vegetable intake. Compared to biomarkers, myfood24^**®**^ overestimated protein intake by less than 5% (86 g/d vs. 82 g/d), and underestimated potassium intake by 36% (3124 mg/d vs. 4267 mg/d). Apart from a higher coefficient in the correlation between EI and TEE in the one-day WFR (7^th^ d) (Table S1), weekly average values assessed by myfood24^®^ had higher CC in the analysed biomarkers.

**Fig. 3.**
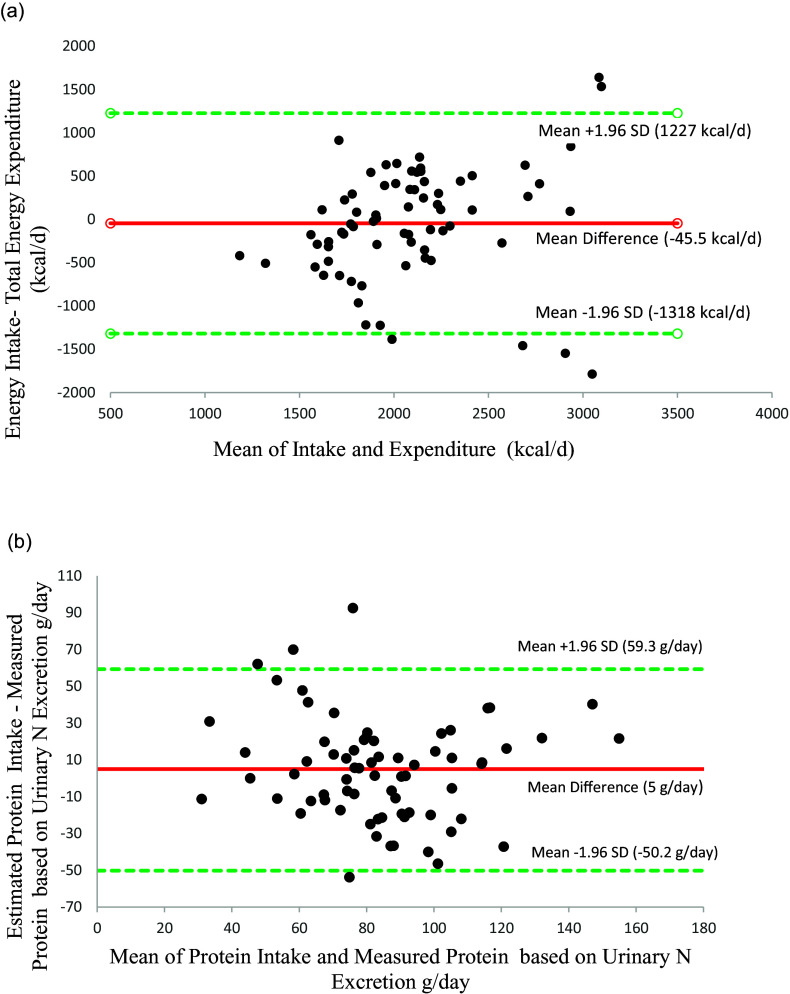
(a) Bland-Altman plot indicating the agreement between the EI based on the 7-day WFR versus the measured TEE among healthy adults (*n* = 71). The middle red line represents the mean difference (–45.5 kcal/d), while the upper and lower dashed lines indicate. the limits of agreement (1227 kcal/d, –1318 kcal/d), corresponding to ±1.96 SD. (b) Bland-Altman plot showing the agreement between the protein intake based on 7-day WFR and the ingested protein as calculated from the 24-hour urinary excretion of N, plotted against the mean of the two measures. The solid line indicates the mean difference between the 2 measures (5 g/d), while the upper and lower dashed lines indicate the limits of agreement (59.3 g/d, –50.2 g/d), corresponding to ±1.96 SD.

Bland-Altman plots indicating the agreement between the EI based on the 7-day WFR versus the measured TEE, and the protein intake based on 7-day WFR and the ingested protein as calculated from the 24-hour urinary excretion of N are shown in Figure 3.

An acceptable correlation (*ρ* = 0.46, *P* < 0.001) was observed between the calculated protein intake based on N excreted in the 24-hour urine samples and the calculated total protein intake from myfood24^®^. Regression analysis showed that protein intake explained 31% of the variance in urinary protein excretion using weekly averages and 30% when adjusted for energy intake using last-day intake (both *P* < 0.001).

Serum folate levels exhibited a strong positive correlation with total folate intake (*ρ* = 0.62, *P* < 0.01) and an acceptable correlation with the dietary intake of fruits and vegetables (*ρ* = 0.44, *P* < 0.01). Total folate intake, inclusive of that coming from supplements, accounted for 43% of the variation in serum folate levels (*P* < 0.001) on a weekly basis and 41% (*P* < 0.001) for the day before V1, while fruit and vegetable intake, major dietary folate sources, explained 29.9% of the variation in total folate intake.

However, the correlation of urinary potassium excretion with dietary fruit and vegetable intake (*ρ* = 0.21, *P* = 0.08) and with dietary potassium intake (ρ = 0.24, *P* = 0.05) was poor. Regression models confirmed that dietary potassium intake explained 1–2% of the variance in urinary potassium excretion (*P* > 0.3) (Supplementary Tables S5a and S5b).

The results after creatinine correction factors were applied are shown in the appendices (Table S3). The correlation between total protein intake and 24-hour urinary protein corrected for creatinine was acceptable (*ρ* = 0.42, *P* < 0.001), which was comparable to the correlation without correction (*ρ* = 0.46, *P* < 0.01). With creatinine correction, the correlation of urinary potassium excretion with dietary fruit and vegetable intake was acceptable (*ρ* = 0.30, *P* = 0.013). Without correction, the correlation coefficient (CC) was lower and not significant (*ρ* = 0.21, *P* = 0.08). The correlation between dietary potassium intake and urinary potassium excretion was acceptable with creatinine correction (*ρ* = 0.26, *P* = 0.03), and slightly lower without correction (*ρ* = 0.24, *P* = 0.05).

The most common types of dietary supplements taken by the participants are summarised in Table S2. Among those using only one type of supplement (*n* = 50), multivitamins were the most frequently reported (*n* = 15). For individuals using 2–4 types of supplements (*n* = 50), calcium, magnesium, and vitamin D (either combined or individually) were the most common (*n* = 35), followed by omega-3 fatty acids (*n* = 19). In cases where individuals used more than four types of supplements (*n* = 14), their intake typically included multivitamins, calcium, magnesium, vitamin D, omega-3 fatty acids, and other combinations of supplements. There was folic acid in the multivitamin tablets taken by 18 of the participants. One participant was using protein powder.

### Reproducibility

The average time interval between the two WFRs was 31.8 ± 6.8 days. With the exception of fish and vitamin D, the Spearman rank correlation coefficients (*ρ*) between the measured variables during WFR1 and WFR2 were statistically significant for all nutrients and food groups (*P* < 0.05), with values ranging from 0.26 to 0.84 (Table 3). This indicates positive associations between measurements, with acceptable correlations for most nutrients. Notably, folate (*ρ* = 0.84), total vegetables (*ρ* = 0.78), potassium (*ρ* = 0.75), dietary fibre (*ρ* = 0.74), and vitamin C (*ρ* = 0.74) demonstrated strong correlations (*ρ* ≥ 0.5).

**Table 3.** Median intake, quartiles, and correlations of nutrients, energy, and food groups from two 7-day WFR (*n* = 69) with significance levels (*P*) of the corresponding Spearman’s rank correlation coefficients and Wilcoxon’s test for paired samples

Nutrients and food groups	7-day WFR 1		7-day WFR 2		Spearman rank correlation (*ρ* (R^2^))	Spearman *P* values	Wilcoxon *P* values
	Median	Q1–Q3	Median	Q1–Q3			
Total veg.* (g/d)	241	159–385	235.0	137–311	0.78 (0.61)	<0.001	0.005
Total fruit (g/d)	106	63–190	102.9	59.4–156	0.62 (0.39)	<0.001	0.04
Meat (g/d)	82.1	47–127	99.0	63.9–130.6	0.73 (0.53)	<0.001	0.08
Fish (g/d)	29.4	13–45	26.4	10.7–40.0	0.30 (0.09)	0.013	0.54
Wholegrain (g/d)	40.3	23–66	38.0	28.9–63.0	0.73 (0.54)	<0.001	0.69
Protein* (g/d)	83.2	72–93	81.0	64.9–92.7	0.64 (0.41)	<0.001	0.19
Energy* (kcal/d)	1992	1675–2371	1956	1626–2317	0.74 (0.55)	<0.001	0.25
Carbohydrate (E%)	42.3	36–46	42.2	37.0–46.2	0.71 (0.51)	<0.001	0.83
Protein (E%)	16.7	15.2–19.2	16.6	14.6–19.3	0.66 (0.44)	<0.001	0.58
Fat (E%)*	35.9	33.1–39.2	35.9	31.9–39.6	0.62 (0.38)	<0.001	0.82
Alcohol (E%)	2.20	0.00–5.22	2.53	0.03–5.08	0.67 (0.44)	<0.001	0.91
Fibre* (g/d)	19.1	15.9–25.4	18.1	14.9–24.8	0.74 (0.55)	<0.001	0.04
Calcium* (mg/d)	902	723–1107	918.0	780–1055	0.71 (0.51)	<0.001	0.16
Vit D* (µg/d)	2.71	1.85–4.28	2.43	1.57–3.71	0.26 (0.07)	0.033	0.17
Vit C* (mg/d)	107.4	78–184	87.7	61.9–144	0.74 (0.54)	<0.001	0.01
Sodium* (mg/d)	2595	2120–3094	2510	2086–3057	0.66 (0.43)	<0.001	0.46
K* (mg/d)	2891	2500–3501	2865	2320–3329	0.75 (0.56)	<0.001	0.01
Folate* (µg/d)	306	196–393	264	191–364	0.84 (0.71)	<0.001	<0.001

* Normally distributed variables after log transformation (Reproducibility): Fat (E%), Protein, energy, vegetable, dietary fibre, calcium, potassium, Vitamin C, sodium, folate. Tot. Veg: Total Vegetable; K: Potassium; Vit C: Vitamin C; Vit D: Vitamin D.

The Spearman correlation coefficients demonstrated strong agreement between identical variables measured in WFR1 and WFR2 (*ρ* = 0.62–0.84, *P* < 0.001). However, Wilcoxon paired tests indicated significantly lower median intake in WFR2 for folate (264 µg vs. 306 µg, *P* < 0.001), total vegetables (235 g vs. 241 g, *P* = 0.005), fibre (18.1 g vs. 19.1 g, *P* = 0.04), vitamin C (87.7 mg vs. 107.4 mg, *P* = 0.01), and potassium (2865 mg vs. 2891 mg, *P* = 0.01). We found no significant differences in median intake of other variables (Table 3). Meat intake was higher during WFR2 compared to WFR1 (99.0 g vs. 82.1 g, *P* = 0.08), though this difference was not statistically significant. Correlations for fish (*ρ* = 0.30, *P* = 0.013) and vitamin D (*ρ* = 0.26, *P* = 0.033) were lower than other nutrients, with no significant differences in their median intakes between WFR1 and WFR2 (*P* ≥ 0.05).

**Fig. 4.**
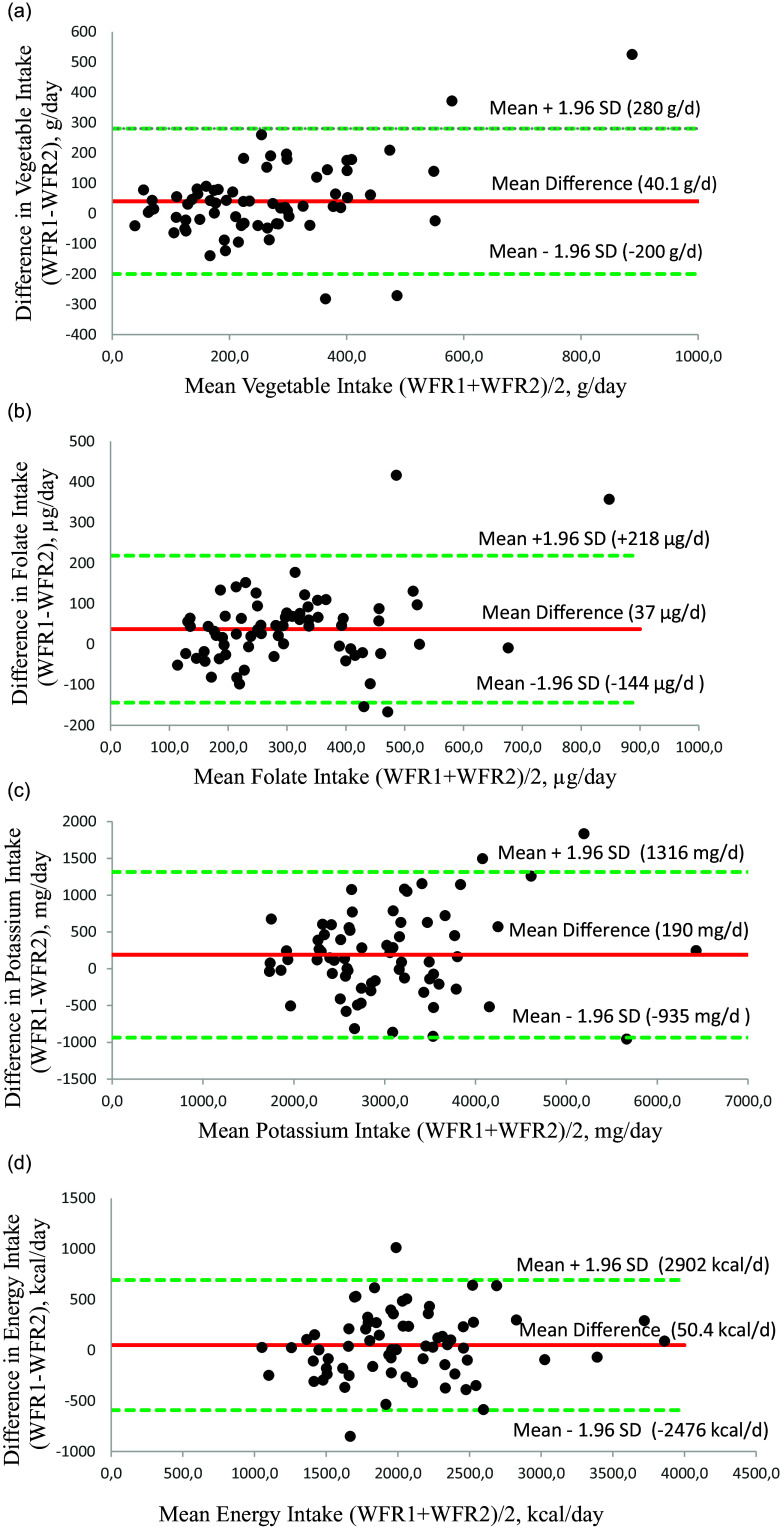
(a) Bland-Altman plot of agreement between total vegetable intake estimated from WFR1 and WFR2. The mean difference is 40.1 g/d, with limits of agreement ranging from –200 g/d to 280 g/d. (b) Bland-Altman plot of agreement between folate intake estimated from WFR1 and WFR2. The mean difference is 37 μg/d, with limits of agreement ranging from –144 μg/d to 218 μg/d. (c) Bland-Altman plot of agreement between potassium intake estimated from WFR1 and WFR2. The mean difference is 190 mg/d, with limits of agreement ranging from –935 mg/d to 1316 mg/d. (d) Bland-Altman plot of agreement between energy intake estimated from WFR1 and WFR2. The mean difference is 50.4 kcal/d, with limits of agreement ranging from –2476 kcal/d to 2902 kcal/d.

Results of agreement between of average weekly intake in the first and the second WFR assessed by the Bland Altman method are presented in Table S4 in the appendices, and Bland-Altman plots for energy, total vegetable, folate and potassium are presented in Figure 4.

These findings suggest that the WFR generally provided consistent and reliable dietary data, with strong correlations for most nutrients and food groups, despite some variability in specific items such as total vegetables and vitamin C.

## Discussion

The biomarkers selected for use in this study are commonly used as dietary biomarkers of the intake of food groups and nutrients. Urinary nitrogen and potassium have previously been used to validate dietary assessment methods,^(42)^ and plasma folate concentration has been identified as a potential biomarker for fruit and vegetable consumption.^(19)^ Overall, we found acceptable to strong positive correlations between the web-based dietary assessment tool and the corresponding dietary intake biomarkers in this healthy, non-representative sample of the Danish population. Our findings are consistent with the findings of previous validation studies. The significant correlation of 0.46 we observed between protein assessed by the N content in 24-hour urine samples and estimated total protein intake was in line with the findings of the NU-AGE study by Ostan *et al.*,^(43)^ which validated dietary intake assessed by 7-day WFR against biomarkers. Hedrick *et al.* ^(44)^ propose that urinary N is a valid biomarker of protein intake, though they warn that a low correlation coefficient of 0.50 may be expected when only a single 24-hour urine collection is used, compared to higher correlations after multiple collections. Hedrick *et al.* also state that there is a potential risk that high protein intakes may be underestimated, and low intakes overestimated when urinary N is used as a marker. In our results, protein intake demonstrated an acceptable predictive value for urinary protein excretion, with approximately one-third of the variance explained, consistent with previous validation studies. Korth *et al.* ^(30)^ investigated correlations of protein intake reported by FFQ versus urinary N used as a biomarker of dietary protein, where they assessed correlations obtained by regressing corrected protein against biomarker protein (6.25 × urinary nitrogen/0.81). They found a CC of 0.31, assessed by Pearson correlation.

We validated measured TEE against estimated energy expenditure from the short IPAQ, a reliable tool for adults.^(32)^ The moderate correlation (*ρ* = 0.27, *P* = 0.02) between EI and TEE, fell within the lower end of the acceptable range (0.20–0.49) and was substantially lower than studies using a wearable sensor,^(45)^ which was beyond our scope.

The correlation between total EI and TEE suggests an acceptable degree of accuracy in self-reported dietary intake reports, supported by 87% of our participants being classified as ARs. However, only 7% of the variation in EI can be explained by variations in TEE at the group level. This suggests that while there was some degree of association between energy intake and expenditure, other factors contributed significantly to the observed variation in energy intake, including daily dietary variations, and limitations of food composition tables.^(44,46)^ Interestingly, our analysis revealed that approximately 14% of the variation in EI could be attributed to variations in REE, underscoring a stronger association of EI with REE as compared to EI with TEE. This may have been due to the instrument we used to assess physical activity, which has previously been shown to have limitations when assessing physical activity in older adults.^(47)^ In the majority of studies comparing IPAQ with objective measurements of physical activity, IPAQ overestimated physical activity by as much as 84%.^(48)^

It is of note that the correlation we observed at the group level may not fully capture individual-level variability, as 93% and 86% of the variation in energy intake remains unexplained by TEE and REE, respectively. We found CC for energy intake and REE comparable to results published by Freedman *et al.*,^(6)^ where they found that de-attenuated CC for women ranged between 0.27 and 0.42. They estimated EI with the mean of three 24HDRs and measured TEE by the DLW method and found protein intake to be underreported by an average of 5%. Foster et al^(24)^ assessed energy intake by an online 24-h dietary recall method (Intake 24) and measured TEE with DLW. They found intra-class correlation coefficients (ICC) of 0.31 for one recall and 0.39 for averaged three recalls. These findings highlight the complexity of energy balance regulation and emphasise the importance of considering individual factors beyond energy expenditure in understanding dietary intake variability. The questionnaire we used to assess physical activity may not comprehensively cover all forms of physical activity, which would contribute to the unexplained variability in energy expenditure.

It is of note that dietary intake on the day immediately preceding the objective measurements explained slightly more of the variation in total energy expenditure (9% vs. 7% for weekly intake), but less of the variation in resting energy expenditure (11% vs. 14% for weekly intake). In a study comparing a FFQ and a recall, Prentice et al^(49)^ found food records to explain 7.8% of biomarker variation for energy; and 22.6% of biomarker variation for protein, and food records had the highest correlations with the biomarkers. Rostgaard-Hansen *et al.* ^(50)^ found correlations between their FFQ and mean of three 24-hour recalls, with a moderate agreement for energy intake (0.30–0.50), similar to our CC of 0.27 (*P* = 0.02) for TEE and 0.44 (*P* < 0.01) for REE. For protein intake, their CCs with urine N ranged between 0.40 and 0.50, comparable to our CCs.

Our present study aimed to examine the relationship between dietary folate intake, including the intake of supplements, and serum folate concentration. Although we observed a significant positive correlation between dietary fruit and vegetable intake and serum folate concentration, and an acceptable correlation between total folate intake and serum folate levels, our results indicate that these sources only partially explain the variations in serum folate levels. This suggests that while dietary folate intake, inclusive of supplementation, significantly influences serum folate levels, other factors, such as the short half-life of folate, genetic predisposition or additional supplements, may play a role.^(19)^ Hedrick *et al.* ^(44)^ demonstrated similar correlations between serum folate concentrations and total folate intake, highlighting plasma folate concentration as a biomarker for fruit and vegetable intake. Our results support this, as we found a significant correlation (*r* = 0.62, *P* < 0.01) between serum folate levels and total folate intake, including the intake of supplements. Furthermore, we found an acceptable positive correlation between serum folate concentrations and dietary fruit and vegetable intake, and folate supplementation (*ρ* = 0.44, *P* < 0.01), suggesting a weaker association compared to total folate intake. Dietary folate intake, inclusive of supplementation, contributes significantly to - but does not entirely explain -variations in serum folate concentrations, which indicates the involvement of additional influencing factors. Folic acid, the synthetic form commonly found in supplements and fortified foods, is generally more bioavailable than natural dietary folate, with an estimated bioavailability around 1.7 times greater.^(51)^ However, the conversion of folic acid to its active form, tetrahydrofolate, takes place in the liver and may be less effective in persons with specific genetic variations, such as MTHFR polymorphisms, resulting in variations in bioavailability or efficacy.^(52)^ The bioavailability of folate from meals can be influenced by the food matrix, which may inhibit or enhance absorption. Although folic acid is more bioavailable, naturally occurring folates in whole foods may have a greater overall health impact due to the additional nutrients and synergistic benefits.^(53)^ The correlation between serum folate and the intake of fruits and vegetables was considered moderate, but this may have been biased by the fact that some of the ingested folate came from sources other than fruits and vegetables.^(54)^ However, the correlation between reported potassium intake from all foods and the potassium excreted in urine achieved barely acceptable correlation coefficients, further confounding the potential of measurements of potassium in urine to conclude on the intake of fruits and vegetables. Moreover, other food groups also contribute to the intake of potassium, limiting the potential of urine potassium excretions as a dietary intake biomarker of fruit and vegetables.

Our results on reproducibility, with CCs ranging from 0.26 to 0.84 across various dietary components, are comparable to previous findings. A study in Danish adolescents by Bjerregaard *et al.* ^(55)^ found acceptable to strong reproducibility for food groups, with mean Spearman correlations of 0.56 and ICCs of 0.61. In their analysis of FFQs completed at baseline and 12 months later, Rostgaard-Hansen *et al.* ^(50)^ found acceptable to strong reproducibility with Spearman rank CCs ranging from 0.44 to 0.72. Based on the Hordaland Health Study population, Sabir *et al.*,^(56)^ found correlations between 0.70 and 0.90 using a web-based FFQ and repeated 24-hour dietary recalls. These consistent findings imply a certain amount of robustness and reliability in dietary assessment methods across varied populations and research contexts at the group level.

Interestingly, while the overall ranking of participants’ dietary intake remained stable, certain nutrients and food groups exhibited greater within-person variability between assessments. For instance, some micronutrients and food groups — including folate, total vegetables, fibre, and vitamin C — showed lower median intake in WFR2 compared to WFR1. This variation may reflect either true day-to-day differences in intake or reporting biases common in self-reported WFRs. Additionally, the weaker correlations for fish and vitamin D, despite no significant differences in median intake, suggest that these dietary components may be more prone to variability in individual consumption patterns, possibly due to their irregular intake frequency.

## Strengths and limitations

One of the key strengths of our study was the use of dietary intake biomarkers, which are assumed to provide a more objective reflection of intake^(57)^ and thereby enhance the accuracy and reliability of a validation study. We implemented a 7-day WFR, providing comprehensive and precise documentation and evaluation of the participants’ dietary intake over an extended period. Supplying all participants with the same brand of calibrated kitchen scales improved the standardisation of the weighing of each food item. Furthermore, all study visits were carefully conducted by trained staff, ensuring consistency and reducing potential bias in data collection.

To ensure the reliability of our urinary biomarker data, we assessed the completeness of urine collections, which is crucial for accurate biomarker analysis. However, standardisation by concentration of urinary creatinine had no noteworthy effect on the measured outcomes. The study design included a 4-week interval between dietary assessments for reproducibility analysis, avoiding the potential confounding effect of seasonal dietary changes.

The use of dietary supplements was recorded and incorporated in our analyses, providing a more accurate representation of nutrient intake. These methodological strengths collectively contribute to the robustness of our findings. While ensuring the weight stability of participants throughout the study, the concept of energy balance was projected to assume that energy intake equalled energy expenditure during the recording period.

Using measured N obtained from 24-hour urine samples in the calculation of REE from indirect calorimetry measurements allowed us to obtain individualised results for each participant.

Among the limitations were the challenges of variability and incompleteness of data in the FCDBs.^(58)^ FCDBs often exhibit significant variability in nutrient values due to differences in species, soil, climate, and food processing methods, and can have incomplete or limited coverage of foods and nutrients, resulting in gaps and inconsistencies in dietary data that hinder accurate estimation of nutrient intake.^(46)^

Based on our validation findings, dietary intake estimates of energy, protein, and folate, together with fruit and vegetables obtained from myfood24^®^ collected as 7-day WFR, provide fairly accurate intake estimates, with some limitations, allowing for reasonable ranking of individuals based on dietary intake. Dietary intake estimates of potassium should be interpreted with caution. Additionally, our reproducibility findings showed strong correlations at group level for total vegetables and wholegrain intake, moderate reproducibility agreements for total fruit and meat, and weak agreement for fish intake. Reproducibility findings of dietary intake estimates at nutrient level (protein, energy and macronutrient distribution (% of energy), dietary fibre, calcium, vitamin C, and sodium) all showed good reproducibility.

## Conclusion

The results of this study demonstrate that myfood24^®^ provides, within certain limits, accurate and reproducible dietary intake estimates in an adult Danish population, confirming feasible accuracy reproducibility in capturing dietary intake. Myfood24^®^ demonstrates reasonable validity and good reproducibility for assessing dietary intake, with moderate correlations to biomarker measures of estimated intake of energy, protein, folate, and fruit and vegetables. Myfood24^®^ also showed strong consistency in reproducibility across most food groups and single nutrients except for fish and vitamin D. While some limitations exist, it remains a useful tool for ranking individuals by intake, particularly in studies focusing on relative comparisons.
