# Validity and reproducibility of a web-based dietary assessment tool: a cross-sectional study in an adult Danish population – ADDENDUM

**DOI:** 10.1017/jns.2025.10027

**Published:** 2025-07-24

**Authors:** Sadime Basak Kisi, Caroline Filskov Petersen, Rikke Sand Andersen, Sidse Ida Ingemann Rasmussen, Alexandr Parlesak, Sine Højlund Christensen, Hanne Lysdal Petersen, Nina Rica Wium Geiker, Mette Friberg Hitz, Inge Tetens

In the original publication of this article, the supplementary material was not included. This has since been added to the paper, and this addendum published.
